# Chitosan Composites Containing Boron-Dipyrromethene Derivatives for Biomedical Applications

**DOI:** 10.3390/ijms24021770

**Published:** 2023-01-16

**Authors:** Aleksander Smolarkiewicz-Wyczachowski, Halina Kaczmarek, Jaroslaw Piskorz, Pawel Nowak, Marta Ziegler-Borowska

**Affiliations:** 1 Faculty of Chemistry, Nicolaus Copernicus University in Torun, Gagarina 7, 87-100 Torun, Poland; 2 Chair and Department of Inorganic and Analytical Chemistry, Poznan University of Medical Sciences, Rokietnicka 3, 60-806 Poznan, Poland

**Keywords:** chitosan, BODIPY, composites, fluorescence, protein adhesion, dye release

## Abstract

The work is devoted to preparing and characterizing the properties of photosensitive composites, based on chitosan proposed for photodynamic therapy. Chitosan films with a 5% addition of two BODIPY dyes were prepared by solution casting. These dyes are dipyrromethene boron derivatives with N-alkyl phthalimide substituent, differing in the presence of iodine atoms in positions 2 and 6 of the BODIPY core. The spectral properties of the obtained materials have been studied by infrared and UV-vis absorption spectroscopy and fluorescence, both in solutions and in a solid state. Surface properties were investigated using the contact angle measurement. The morphology of the sample has been characterized by Scanning Electron and Atomic Force Microscopy. Particular attention was paid to studying the protein absorption and kinetics of the dye release from the chitosan. Adding BODIPY to the chitosan matrix leads to a slight increase in hydrophilicity, higher structure heterogeneity, and roughness, than pure chitosan. The presence of iodine atoms in the BODIPY structure caused the bathochromic effect, but the emission quantum yield decreased in the composites. It has been found that BODIPY-doped chitosan interacts better with human serum albumin and acidic α-glycoprotein than unmodified chitosan. The release rate of dyes from films immersed in methanol depends on the iodine present in the structure.

## 1. Introduction

Despite enormous progress in the diagnosis and treatment of cancer in recent decades, cancer is still difficult to cure. New methods used in cancer treatment include immuno-oncology, gene therapy, molecular targeted (personalized) therapy, and the photodynamic techniques [1,2,3]. The contemporary strategy for combating these dangerous diseases is based primarily on developing diagnostic methods, designing modern drugs and ways of precisely delivering them to the affected places.

A large group of currently used active medicinal substances consists of low-molecular organic compounds characterized by serious disadvantages—rapid metabolism, insufficient drug resistance, and lack of selectivity in numerous cases. It results in their rapid excretion from the body, inadequate distribution, and low therapeutic effectiveness. Therefore, an intensive search for carriers that could increase their potency is underway. Polymers, especially biopolymers, are promising materials that, when combined with bioactive substances, create conjugated systems capable of gradually releasing the drug at the target site, due to metabolic processes [4]. In addition, the polymer matrix can play a protective role in preventing the premature release or degradation [5,6].

Among the many macromolecular compounds currently used in biomedicine, chitosan (**CS**) is distinguished. It is a biopolymer consisting mainly of D-glucosamine linked by a β-(1→4) bond and a minor part of N-acetyl-D-glucosamine units that are the remainder of the chitin substrate. Due to various functional groups (OH, NH_2_, CH_3_CONH_2_), chitosan has unique properties and is capable of many interactions (hydrogen, hydrophobic, and ionic bonding) and chemical reactions. Moreover, it is biocompatible, biodegradable, non-toxic, antimicrobial, and readily available. It allows for the understanding of its increasing application, not only in medicine (e.g., in surgery, tissue engineering, drug delivery systems, and wound healing), but in many other industries (packaging, cosmetics, agriculture, and veterinary) [7,8,9].

The discovery of the photodynamic reaction in the early 1900s contributed to the development of photodynamic anticancer therapy (PDT), which is now the subject of intense research [10,11,12,13]. This method involves giving the patient a photosensitizer, then exposing it to light and causing a chemical reaction that produces the reactive oxygen species, leading to apoptosis of the cancer cells. A particular advantage of PDT is its high selectivity and the absence of side effects, unlike in chemotherapy, radiotherapy, and surgery. Properly selected photosensitizing compounds, characterized by the specific absorption of radiation in the visible range and ability to fluorescence, as well as exposure conditions (presence of oxygen, dose), play a crucial role here [14]. A novelty in the PDT method is its combination with nanotechnology and nanomaterials. Such examples are polymeric nanocarriers loaded by a photosensitiser, hybrid nanoparticles, or a photoactive compounds encapsulated in a polymer shell that contribute to the proper administration and positive accumulation of the drug in tissues, with a higher efficiency of the formation of the reactive oxygen species and the increase the cancer cells death with no effect in the neighboring cells, as well as an improved stability [15,16,17,18].

However, the effectiveness of the PDT method still needs to be improved upon by an intense search for new effective photosensitizers and application methods. Over several decades, there have been many reports of such compounds with a potential use in PDT, including boron-dipyrromethene and its derivatives (called BODIPY dyes), reviewed recently by M. Poddar and R. Misra [19] and E. Antina et al. [20]. The structure of these compounds is based on the connection of two heterocyclic pyrrole rings through a difluoroboryl unit, resulting in a three-ring system with six resonating pairs of π electrons. The BODIPY core is flat and prone to chemical modification (e.g., substitution in λ, β or *meso* position), leading to changes in the photochemical properties.

There are several examples demonstrating the latest achievements in this field, e.g., BODIPY functionalized with lactose [21], phosphorylated BODIPY [22], diketopyrrolopyrrole-aza-BODIPY hybrids [23], N-BODIPY containing a para-nitrophenyl group at the *meso* position and a diamino-boron substituent with two tosyl pendant moieties [24], trifluoromethyl-substituted (i.e., with electron-withdrawing groups) and methoxy-substituted (with electron-donating groups) dyes [25].

The purpose of this study was to study the photosensitive composites based on chitosan (**CS**) containing two dipyrromethene boron difluoride derivatives (BODIPY dyes: **I** and **H**) with N-alkyl phthalimide substituent at the meso position. The dye marked **I**, contains two iodine atoms in positions 2 and 6 instead of hydrogen atoms (present in the **H** structure). In this way, it is possible to assess the influence of heavy atoms in BODIPY-type chromophores on the properties of chitosan modified with these dyes. It should be added that substituent in position 8 usually causes the desirable blue emission.

The spectroscopic and surface properties and the morphology of the obtained systems were characterized. Special attention was paid to the ability to interact with serum proteins by the composites and the release of dyes from chitosan films, which is relevant in biomedical applications.

The synthesis of applied modifying dyes: boron-dipyrromethene derivatives with substituted N-alkyl phthalimide groups has been previously described by Piskorz et al. [26]. The photodynamic antimicrobial activity of these compounds and their ability to generate singlet oxygen has also been proven in this work.

## 2. Results and Discussion

### 2.1. General Remarks and Visual Observation of Chitosan-BODIPY Composites

**Figure 1:**
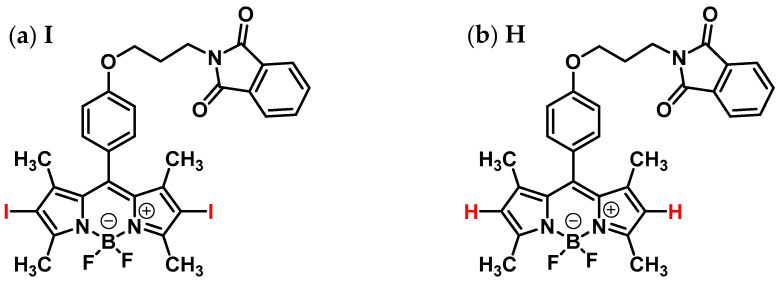
Chemical structure of BODIPY dyes used for chitosan modification: (**a**) 1,3,5,7-tetramethyl-8-{4-(3-phthalimide)propoxy]phenyl}-4,4-difluoro 4-bora-3a,4a-diaza-s-indacene (**I**); (**b**) 2,6-diiodo-1,3,5,7-tetramethyl-8-{4-(3-phthalimide)propoxy] phenyl}-4,4-difluoro-4-bora-3a,4a-diaza-s-indacene (**H**). Red atoms indicate the difference in the chemical structure of both compounds.

Two different BODIPY dyes were introduced into the chitosan matrix (Figure 1).

These dyes differ in the presence of two iodine or hydrogen atoms in positions 2 and 6 of the BODIPY cyclic structure.

**Figure 2:**
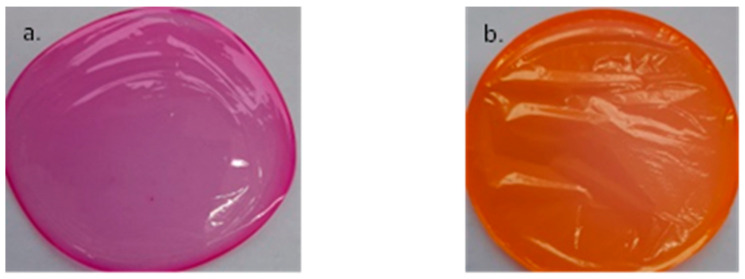
Photo of chitosan film (**a**) with 5% addition of **I** and (**b**) with 5% addition of **H** dyes.

The presence of the phthalimide substituents in the BODIPY structure can give the dye additional biological activities, including antimicrobial, anti-inflammatory, antinociceptive, anti-convulsant, and analgesic effects [26,27]. Moreover, heavy iodine atoms in the dye core increase the singlet oxygen production, which improves the therapeutic effect in treating tumors [28]. Introducing 5% wt BODIPY compounds into a chitosan solution and obtaining solid films by casting and evaporating solvents is uncomplicated. The composites obtained by this method were visually homogenous, transparent, and relatively flexible. The sample obtained with 5% **I** was deep purple, while the mixture with 5% **H** was intense orange (Figure 2). It can be added that the chitosan film itself was colorless.

### 2.2. Infrared Spectroscopy

**Figure 3:**
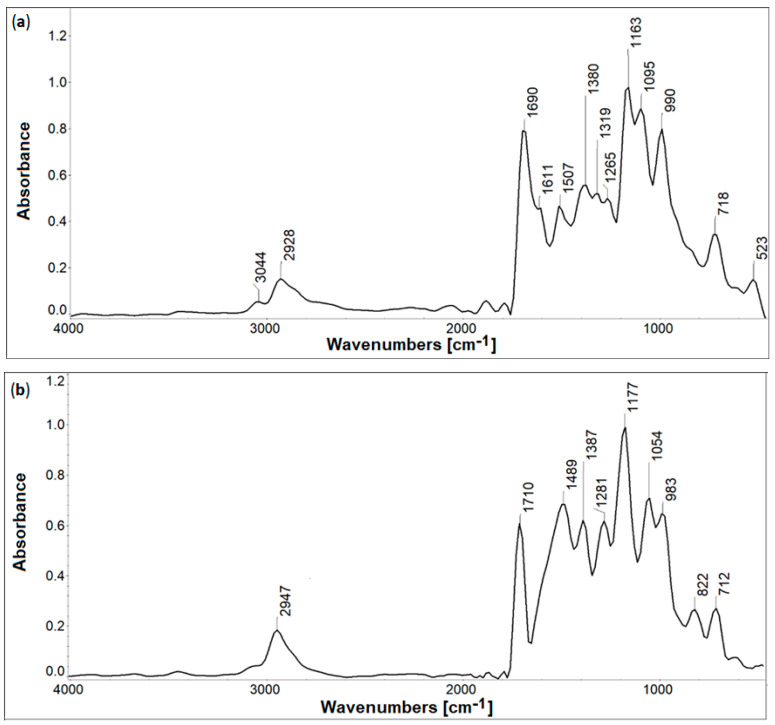
FTIR-ATR spectra of BODIPY: (**a**) **I** and (**b**) **H**.

FTIR-ATR spectra of applied **I** and **H** dyes are presented in Figure 3a,b. In both spectra, similar absorption bands appear, with slight shifts, as shown in Table 1. The band at 2928/2947 cm^−1^ is attributed to C-H stretching vibrations in CH/CH_2_/CH_3_ groups. The weak band of C-H in the aromatic rings is also seen at 3044 cm^−1^ and 3083 cm^−1^ in **I** and **H**, respectively.

The carbonyl band occurs at 1690/1710 cm^−1^, C=C in the aromatic rings at 1507/1489 cm^−1^, C-O at the range of 1100–1400 cm^−1^, and C-C at 990/983 cm^−1^. The peak at 1380/1387 cm^−1^ may be due to the overlapping vibrations of the C-H (deformation) and B-N groups (stretching, in-plane) [29,30,31]. A band at 523 cm^−1^ in the **I** dye spectrum can be attributed to C-I stretching vibration [32]. The fingerprint region at 1000–1500 cm^−1^ is particularly rich in multiple bands, which is typical for such dyes. In this range, the main differences between both compounds appear.

**Figure 4:**
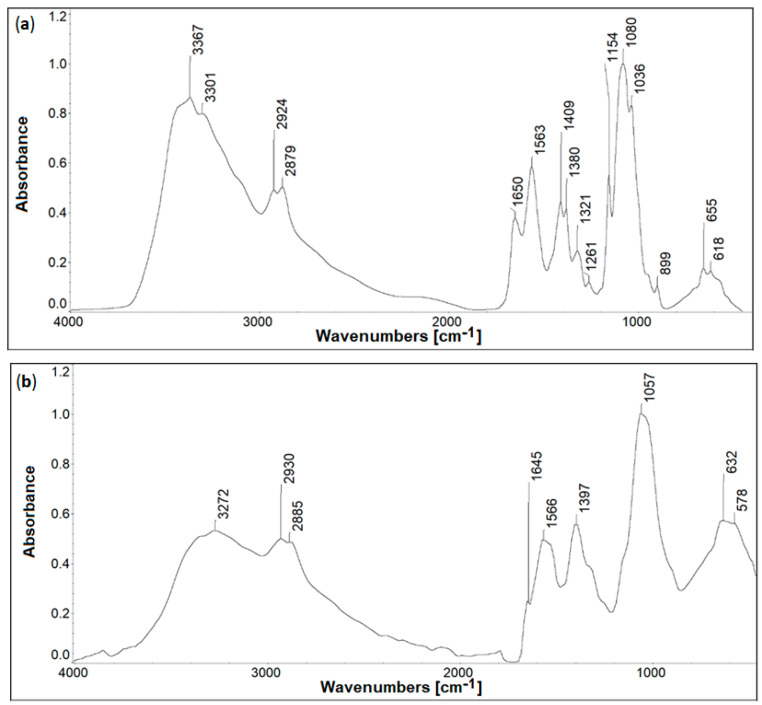

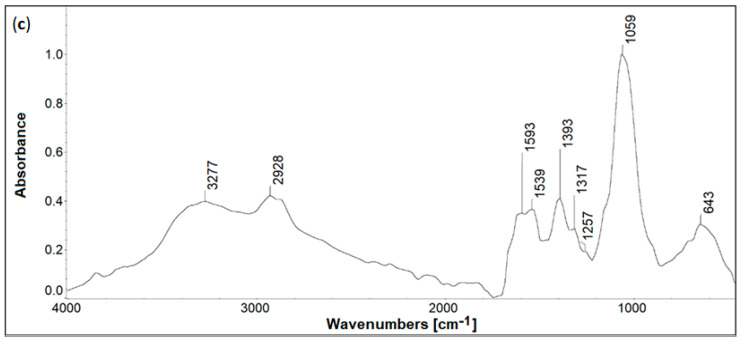
FTIR-ATR spectra of chitosan (**a**) and **CS-I** (**b**) and **CS-H** (**c**) composites.

Compared to pure, unmodified chitosan, FTIR spectra of chitosan-BODIPY composites show negligible differences (Figure 4a–c). In these three spectra, there are bands characteristic of polysaccharides, the main ones are: hydroxyl (with overlapping amine) at 3000–3600 cm^−1^ range, C-H at ~2880–2930 cm^−1^, amide at ~1500–1700 cm^−1^, C-O-C at ~1000–1200 cm^−1^. The bands typical for the aromatic and heterocyclic rings in dyes are covered with intensive chitosan bands. However, the band arm at 578 cm^−1^ in **CS-I** spectrum indeed arises from the presence of the B-N group. The C=O amide I band, visible in CS at 1650 cm^−1^, becomes the branch (at cm^−1^) of the stronger amide II bands at 1566 cm^−1^ and 1593 cm^−1^ in the **CS-I** and **CS-H** spectrum, respectively. A detailed interpretation of the bands is presented in Table 2.

**Scheme 1:**
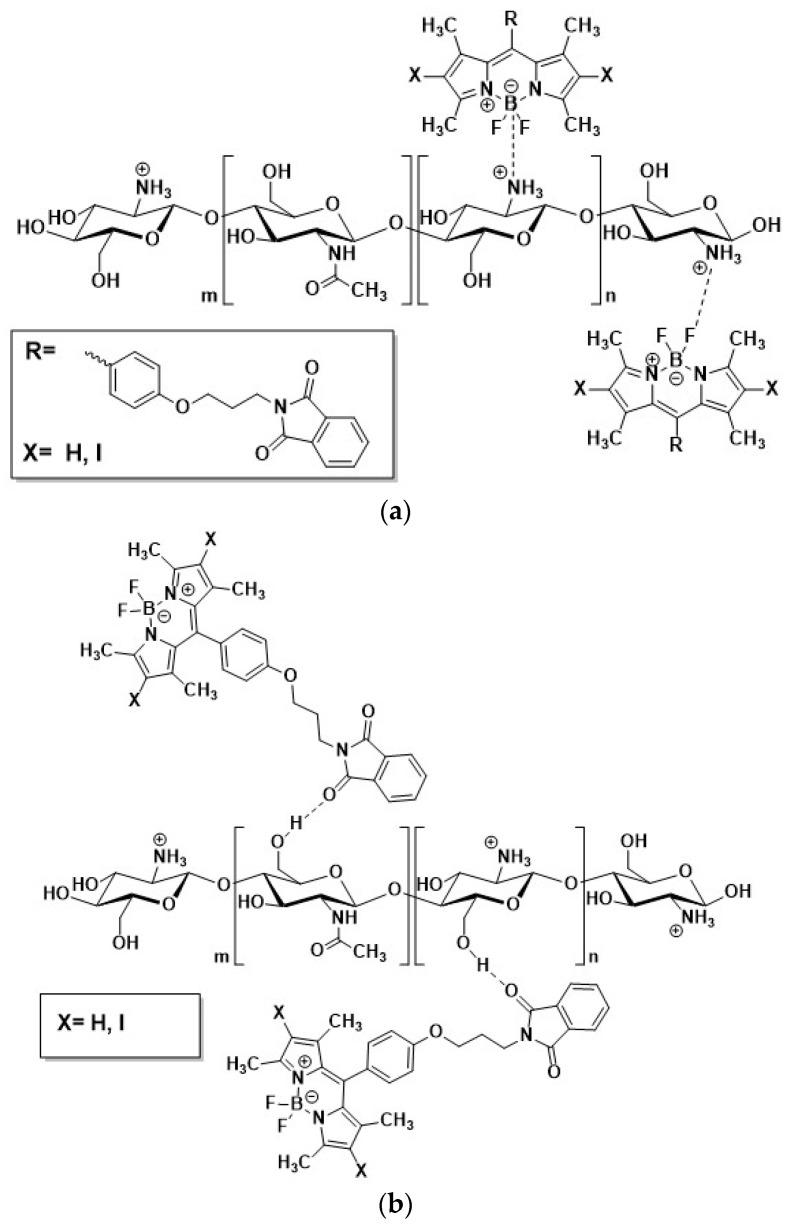
Possible interactions between BODIPY and chitosan groups: -B^−^…^+^NH_3_^−^ (**a**) and –C=O…H-O…(**b**).

Generally, the main detected differences appear in the range corresponding to the C=O and C-O vibrations and in the fingerprint region (600–1400 cm^−1^). The disappearance of the weak band at 897 cm^−1^ in the spectra of the chitosan doped with dyes was also observed. These changes indicate the intermolecular interactions between chitosan and **I** or **H** dye. The dipole-dipole interactions are particularly probable due to the functional groups containing polarized chemical bonds: N-H, O-H, C=O in **CS**, C=O, B-F in **H** and **I** (polarity of C-I bond in **I** is negligible because of the slight differences between the electronegativity of carbon and iodine). Dipole-ion interactions between protonated amine groups (in **CS**) and anionic boron atoms (in dyes) cannot also be excluded (Scheme 1).

### 2.3. UV-Vis Absorption Spectroscopy

**Figure 5:**
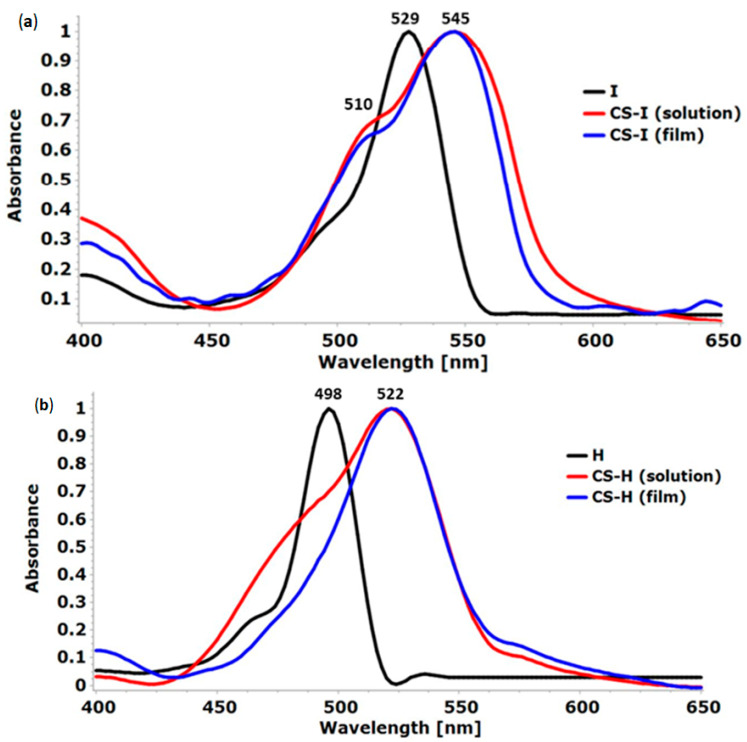
Normalized absorption spectra of BODIPY dyes (in acetone solutions) and chitosan composites (in solutions and solid films): **I** and **CS-I** (**a**); **H** and **CS-H** (**b**).

Electronic spectra of **I** and **H** dyes and CS-BODIPY indicate the strong bands in the visible range of 450–600 nm, resulting from the transitions of π electrons to the π* excited state (Figure 5, Table 3). The absorption band in the composite spectra, both in solution and in the form of solid films, show a bathochromic effect in relation to the spectra of the dyes themselves, i.e., maxima are shifted towards longer wavelengths by 16 nm and 76 nm in **CS-I** and **CS-H**, respectively. Interestingly, no differences in maximum location are found in the spectra of films and solutions; thus, the solvents’ effect seems negligible.

However, the significant broadening of these bands in **CS-I** and **CS-H** spectra is observed, compared to the related bands in **I** and **H** dye absorption spectra. The broadening is higher in the case of solutions than that in solid **CS-I** and **CS-H** spectra. It is caused by the interactions of solvents with dye-doped chitosan. As recently shown [36], the aromatic substituent in BODIPY also causes the broadening of the absorption band in aqueous solutions.

The asymmetric shape of absorption bands with the formation of the left-side branch, indicates the possibility of the dye aggregation. This asymmetry is very weakly marked in the spectrum of the **CS-H** film. The red shift of absorption bands in **I** and **CS-I**, compared to **H** and **CS-H**, is caused by the presence of heavy iodine atoms in the BODIPY structure.

### 2.4. Fluorescence

**Figure 6:**
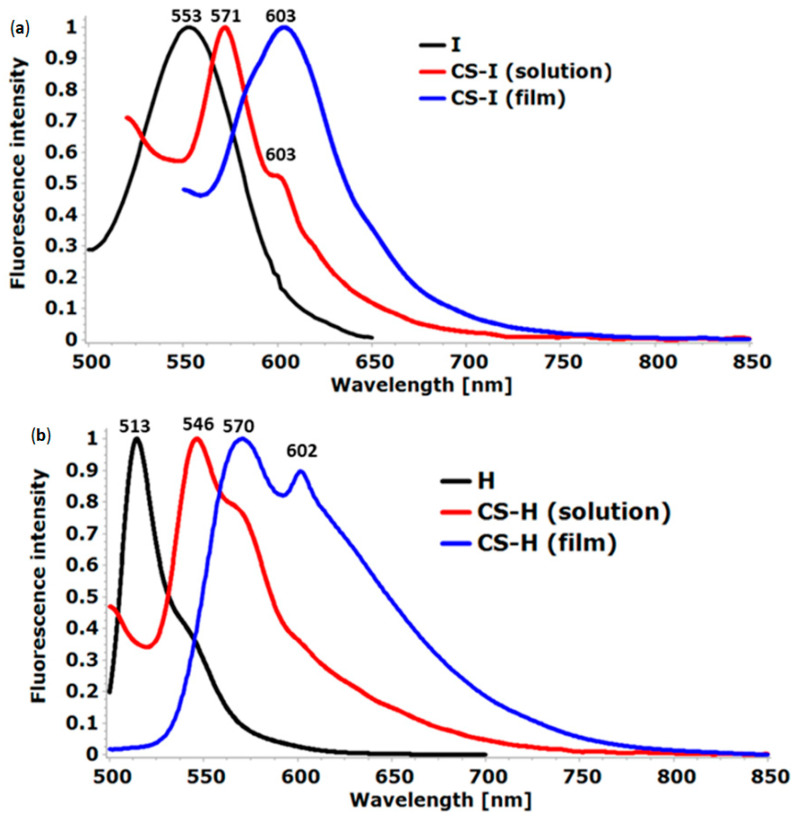
Normalized emission spectra of **I** (**a**) and **H** (**b**) dyes, as well as **CS-I** and **CS-H** in a solution or solid state.

Fluorescence spectra for BODIPY dyes and CS-dye composites in solutions and solid state are shown in Figure 6. As can be seen, the emission maxima shift to longer wavelengths for the **CS-I** and **CS-H** solutions and films. Stokes shifts are particularly high in composite solid films—58 and 80 nm in **CS-I** and **CS-H**, respectively (Table 3).

As it is known from the literature, dyes of the BODIPY type are prone to aggregation [36,37,38]. The observed redshift suggests the formation of J-type aggregates in the chitosan matrix and solution. Due to the four methyl groups, the phenyl ring in the meso position does not rotate and lies in the same plane as the BODIPY, which would allow the formation of such aggregates. However, the phthalimide substituent is connected with the phenyl ring by a flexible propoxy spacer, enabling it to assume different conformations (with different degrees of twisting, relative to the BODIPY core). This makes it difficult to build aggregates. Thus, two opposing effects influence the structure of the chitosan-dye systems.

It was reported earlier that such a bathochromic shift of the band in the emission spectra, is profitable from the point of view of biomedical applications, as it leads to less absorption and, at the same time, a deeper penetration of light into tissues [39].

The intensity and quantum yield of fluorescence are relatively low. The sample without iodine is characterized by higher Φ_F_ values than the corresponding specimens containing iodine atoms, indicating the heavy atom’s effect. The most intensive fluorescence (in acetone solution) exhibits **H** dye (Φ_F_ = 19.4%), whereas the **CS-I** film showed the highest quenching of radiation emission. As previously stated, fluorescence suppression is caused by efficient intersystem crossing (ISC), leading to a drop in the population of molecules in the singlet excited state [26,40]. However, the triplet state that arises from the ISC process promotes the formation of singlet oxygen or other reactive species [41]. Another reason for the decrease in the BODIPY fluorescence intensity in chitosan systems may be the above mentioned particle aggregation [36]. Moreover, fluorescence suppression was also attributed to water present in the solution mixture [36].

### 2.5. Contact Angle Measurements

To determine the surface free energy of chitosan film and its composites with BODIPY compounds, the contact angle was measured with two test liquids of different polarities: glycerin and diiodomethane (Table 4). It is mentioned that glycerin was chosen instead of H_2_O as the polar liquid [42] since the water has recently been found to be unsuitable for testing biopolymer surfaces [43]. The contact angle of the water changes rapidly during the measurement, and the drop spreads over the biofilm surface [43].

Based on the mean values of the contact angles and the calculated surface free energy (SFE), it can be stated that the surfaces of all obtained materials are hydrophobic (Table 4). This is also confirmed by the values of the SFE(D) dispersion component, which is much greater than the polar component SFE(P) in each studied specimen. Therefore, weak dispersion interactions, resulting from electron density fluctuations, dominate in the surface layers of the samples despite the presence of polar hydroxyl groups in the chitosan structure. Such surfaces are characterized by a poor wettability by water. Other authors obtained similar results for chitosan [44]. The hydrophobicity of this polysaccharide is explained by the presence of the hydrophobic nature of the CS backbone [45] or by the impurities, present even in small amounts [46]. It also depends on the deacetylation degree because the amino groups are hydrophilic, as opposed to the acetyl groups [9].

Chitosan films with the addition of **I** or **H** dye exhibited a slightly higher hydrophilicity than the un-doped chitosan. The location of ionic fragments of BODIPY and fluorine atoms at the top layer of the sample can explain it. In addition, both dyes have an 8-position substituent that contains two electronegative oxygen atoms and one nitrogen in the phthalimide ring, and an additional one oxygen atom in the propoxyphenyl linker. In the case of **CS-I**, iodine atoms also influence an increase in polarity (8.64 mJ/m^2^). However, the slight difference between the electronegativity of iodine and hydrogen (2.20 and 2.66, respectively) does not explain the observed increase in the polarity of **CS-I**. The reason is probably because of the different conformation of the dyes in **CS-H** and **CS-I**, where a large I atom can cause additional steric hindrances (the covalent radius of **H** and **I** is 25 and 40 pm, respectively).

Results show that the surface free energy values of samples are within the range at which cell adhesion is possible (20–30 mJ/m^2^) [47]. Thus, the potential application of the obtained materials as a drug carrier in the form of patches can be predicted.

### 2.6. Morphology (SEM and AFM)

**Figure 7:**
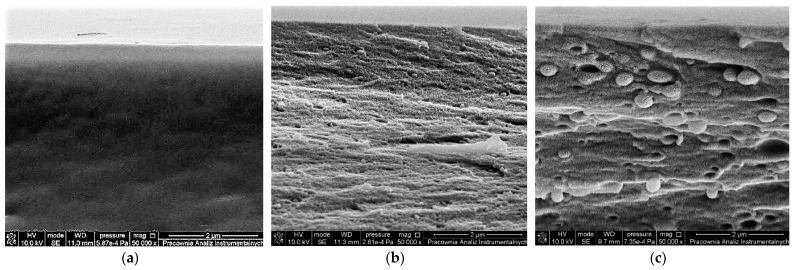
SEM images of a cross-section of chitosan (**a**), **CS**-**I** (**b**), and **CS**-**H** (**c**) films at 50,000× magnification.

SEM images of the chitosan cross-section (Figure 7a) indicate its compact homogeneous structure without any special details, in contrast to the samples with the addition of BODIPY dyes (Figure 7b,c). Apparent spherical inclusions with dimensions of approx. 0.2–0.5 μm can be observed in the **CS**-**H** sample (Figure 7c), where simultaneously, the roughness parameters take the highest values (Table 5). It can be explained by the precipitation of the BODIPY dye in a chitosan matrix during solvent evaporation and film molding. Sample **CS**-**I** presents a different image of the internal structure, which is also heterogeneous, but the scattered particles are much smaller than those in **CS**-**H** (Figure 7c).

**Figure 8:**
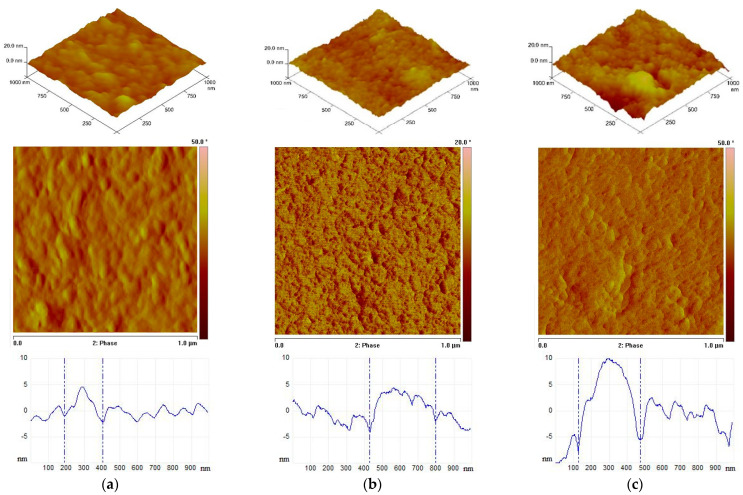
3D-AFM (upper panel), phase AFM images (middle panel), and cross-sections (bottom panel) of chitosan (**a**), **CS-I** (**b**), and **CS-H** (**c**) films for a 1 × 1 µm^2^ scan area.

AFM allows for the observation of the surface topography and the assessment of the surface roughness, which, apart from SEP, also affects the adhesion to other materials, e.g., tissues. The surface topography of the samples is influenced by various factors, including the chemical structure of the compound, homogeneity of the composition, and the presence of inclusions or impurities. However, in the case of the tested samples, the differences in the surface structure are not very large (Figure 8). The surface of chitosan films shows a nodular structure but is relatively smooth, which is also observed by other authors [44,48]. The **CS-H** sample exhibits larger aggregates randomly dispersed in the chitosan matrix. This is also seen in the cross-section of this sample (Figure 8c, bottom).

Furthermore, the determined roughness parameters are low for all studied samples (Table 5). R_q_, R_a_, and R_max_ values for **CS** and **CS-I** are similar and are only somewhat higher for **CS-H**. This trend is observed for smaller (1 × 1 µm^2^) and larger (10 × 10 µm^2^) scan areas. Thus, incorporated BODIPYs have a negligible effect on the surface geometry. It indicates that the precipitated dye particles are rather below than on the surface, as also shown by the SEM pictures. This can be an advantage because the biopolymer layer plays a protective role in such cases, e.g., against the destructive environmental or chemical factors. Moreover, it is a reason for the weakening of fluorescence in solid composites. Relatively high R_max_ values are caused by local structural imperfections or impurities in **CS**, and in the case of **CS-I** and **CS-H** systems—also by the presence of precipitated dye particles.

A low surface roughness indicates that the condition required for measuring the contact angle, i.e., R_a_ < 0.5 µm [49], is fulfilled.

### 2.7. Protein Adsorption

Non-toxic and biocompatible chitosan is a good material for drug delivery [50,51]. CS as a cationic biopolymer can be mucoadhesive through interaction with the mucosa of living organisms [52]. Due to the presence of protonated amino groups, ionic bonds can be formed with the sialic acid residue in the glycoprotein present in the mucosa. As a result of the adsorption of the biopolymer carrier, biologically active substances are released locally on the surface of the affected skin, and the treatment is effective. However, modified chitosan, e.g., in combination with a drug, may show other, sometimes unfavorable properties, a lower availability of positively charged amino groups, and thus a reduced adsorption capacity [51]. Therefore, studying the adsorption of proteins on biopolymer materials intended for therapeutic purposes is extremely important.

**Figure 9:**
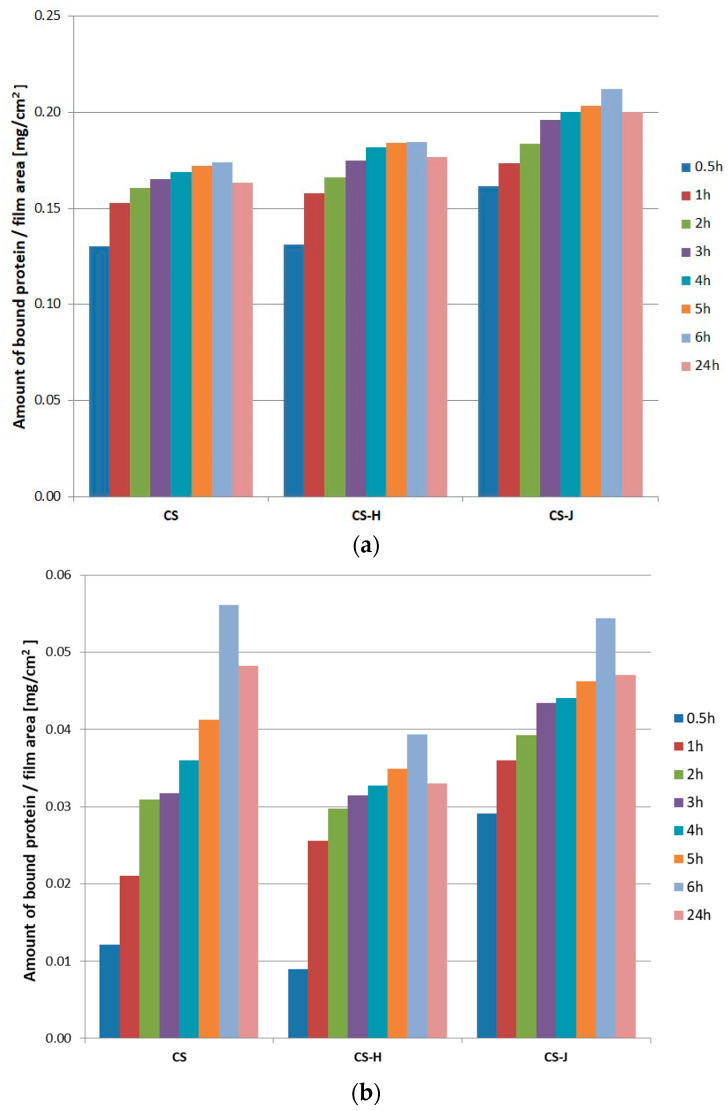
The amount of bound HSA (**a**) and AGP (**b**) proteins (in mg per 1 cm^2^ of biofilm) of the tested samples: chitosan, chitosan doped with **I** and **H** dyes.

Two types of protein: human serum albumin (HSA) and α-acid glycoprotein (AGP), have been used to determine the interaction with BODIPY-modified chitosan films. The research results indicate that human serum albumin interacts better with chitosan films doped with dyes than with pure chitosan (Figure 9a). The increase in adsorbed HSA protein was observed up to 6h of incubation. However, after 24 h of the test, desorption of HSA from the composites was noted. This is convenient, due to the fact that the protein-bound drug should be released as the plasma concentration of the free fraction of the drug decreases. The reversible binding of plasma proteins by the resulting composites is therefore promising, in terms of pharmacological properties. The chitosan composite (**CS-I**) with the addition of dye **I** was the best for the adsorption of human serum albumin, among those three materials.

Sample **CS-I** also shows a greater ability to adsorb acidic α-glycoprotein (AGP) than **CS** and **CS-H** (Figure 9b) in the initial incubation period (up to 6 h). In this test, similarly to the HSA adsorption study, the desorption of AGP adsorbed on the materials was noticed after 6 h of incubation.

The chitosan used in this study has a relatively low molecular weight, which, as is known, allows not only for the external interaction with the target organism, but also has the ability to penetrate into tissues, resulting in intracellular interactions [9]. At the same time, in addition to the action of the photosensitive compound introduced, the biological effect of chitosan itself on infected skin areas can be enhanced. Attachment to proteins in the microbial cell leads to changes in their configuration and consequently to the disturbance of their vital functions. Moreover, as it has recently been reported, that pristine chitosan also exhibits anti-tumor activities [9], further enhancing the proposed systems’ effectiveness.

### 2.8. Kinetics of the Dyes’ Release

Another important feature of materials with potential medical applications, apart from the adsorption capacity, is the gradual release of the applied drug. Chitosan, thanks to its unique features (including biocompatibility, adhesion properties, cationic nature, and non-toxicity), has already been proposed as a drug carrier [53] and also as a photo-controlled delivery system [54].

**Figure 10:**
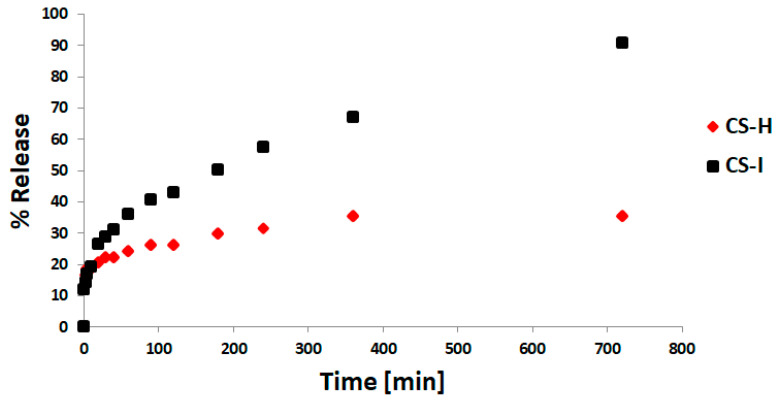
Kinetics of the release of dyes **I** and **H** from the chitosan film.

The release kinetics of BODIPYs from the studied chitosan matrix is shown in Figure 10. As can be seen, the **H** dye is released much more slowly than the **I** dye. The concentration of this compound in the extract was noticeable after 5 min of the study, but the increase in its amount over time was relatively slow. Following 12 h of the study, its released amount constituted 35% of the mass of the **H** dye introduced into the sample. The **I** dye is released from the chitosan film much faster. Its release efficiency after 12 h is already 90%. The difference in the release rate of these two similar compounds is undoubtedly due to the presence of large iodine atoms in **CS-I**, which may constitute steric hindrances in intermolecular interactions. This probably makes it difficult for **I** dye molecules and **CS** macromolecules to move closer to each other, and therefore, a looser structure is formed. Thus, this dye is well dispersed in the chitosan matrix and more labile. Moreover, the lack of heavy atoms in the structure of the **H** dye causes the formation of larger dense clusters in the solid state (confirmed by SEM microscopy), from which the dye is released more slowly. Moreover, the particles of this dye can partially penetrate the chitosan random coils, from which they are eluted for a longer period of time by methanol.

It is worth emphasizing that the released BODIPY dye can generate singlet oxygen under the influence of radiation with a wavelength of 615 nm [55]. The determined quantum yields of singlet oxygen generation Φ_O2_ are 0.48 and 0.06 for **I** and **H**, respectively. These results indicate a heavy atom effect, i.e., a significant increase in Φ_O2_ for a compound containing iodine atoms. Given the greater potential of compound **I** for photodynamic therapy and the fact that it releases from the chitosan composite much better than the less active compound **H**, it can be said that this chitosan-based material has potential as a new form of photosensitizer for this therapy.

### 2.9. Mechanical Properties

To determine the mechanical strength of the obtained films, tensile tests were carried out. These tests allowed us to record stress–strain curves and determine the breaking stress, ultimate elongation, and Young’s modulus, which characterizes the material’s elasticity. Young’s modulus was calculated as the tangent of the angle in the initial, rectilinear course of the relationship where Hooke’s law is fulfilled. The results from this analysis are presented in Table 6.

As can be seen, the addition of 5% BODIPY to the chitosan matrix deteriorates the mechanical properties, which is the result of a poor miscibility of the components. The drop in breaking stress is most significant in **CS-H**, but Young’s modulus takes a relatively large value (767 MPa), indicating a good elasticity. In turn, the **CS-I** sample shows the largest decrease in Young’s modulus, but the breaking stress changes are relatively minor, compared to this parameter in the chitosan alone. Low elongation values are typical for non-plasticized chitosan materials [56,57]. Changes in this parameter are relatively small in both dye-modified chitosan samples.

However, it can be stated that the obtained mechanical properties are sufficient for the proposed potential applications in the biomedical industry, e.g., in the form of patches or dressing materials containing photosensitizers.

## 3. Materials and Methods

### 3.1. Materials

Chitosan of a low molecular weight (50,000 g/mol) and deacetylation degree of 75–85% was supplied by Chemat (Konin, Poland); acetic acid (99.5–99.9%) by Avantor Performance Materials Poland; solvents (methanol, acetone) and salt hydrates—monosodium phosphate, NaH_2_PO_4_·2H_2_O, and disodium phosphate, Na_2_HPO_4_·12H_2_O, were purchased from Chempur (Piekary Slaskie, Poland). Glycerin, diiodomethane, and proteins—human serum albumin (HSA) and α-acid glycoprotein (AGP) were provided by Sigma-Aldrich. Chitosan and the other reagents (of high purity) were used without further purification.

Two BODIPY chromophores (**I** and **H**), synthesized in a two-step process, described previously in detail [26], were supplied by J. Piskorz from Poznan University of Medical Sciences in Poland. The chemical structures of these dyes have been confirmed by mass spectrometry (HRMS) and ^1^H and ^13^C NMR spectroscopy. The chemical formulae, systematic names, and abbreviations are shown in Figure 1.

### 3.2. Preparation of the Chitosan Composites with BODIPY Dyes

One g of chitosan was dissolved in a 250 mL flask containing 100 mL of 1% acetic acid. Then, 50 mg of the dye (**I** or **H**) previously dissolved in three drops of acetone was added. The mixture was stirred until the compound was completely dissolved. Homogeneous solutions were poured onto Petri dishes and placed on a leveled table. A chitosan film alone without adding a dye was also prepared as a reference. Following the evaporation of the solvent, the films were dried in the dark. The film thickness was **CS**-0.026 mm, **CS-I**-0.012 mm, and **CS-H**-0.032 mm. The abbreviations used in study are **CS-I** and **CS-H**.

### 3.3. Research Methodology

#### 3.3.1. FTIR and UV-Vis Absorption Spectroscopy

Infrared spectra were obtained using Spectrum Two (Perkin Elmer, Waltham, MA, USA) with an ATR attachment containing a diamond crystal (angle of incidence 45°, one reflection). The range was 4000–400 cm^−1^, the scanning speed was 0.2 cm/s, and the number of scans was 32.

The UV-Vis spectra were recorded with a Shimadzu UV-1800 double–beam spectrophotometer (Kioto, Japan) in the visible range of 400–800 nm in quartz cuvettes with an optical length of 1 cm. To determine the wavelength at which the maximum absorption by the dyes used occurred, they were dissolved in acetone, diluting the obtained solutions so that their absorbance was close to 1. The final concentration was 3.125 × 10^−3^ M for both dyes. The spectra of chitosan composites in the solid thin films were also measured.

#### 3.3.2. Fluorescence and Quantum Yield Determination

The emission spectra of the samples in the solutions (**I**, **H**—in acetone, **CS-I**, **CS-H**—in 1% CH_3_COOH + acetone) and in the solid films (**CS-I**, **CS-H**) were recorded with the use of the Jasco FP-800 spectrofluorimeter (Tokyo, Japan) in the visible range of 400–900 nm. The dye concentrations in the solutions were chosen so that the emission intensity was within the scope of 0.1–0.2. The concentration obtained by diluting the solutions was 1.5625 × 10^−4^ M and 3.125 × 10^−4^ M for **I** and **H**, respectively.

Quantum fluorescence yields were determined using the Jasco FP-800 spectrofluorimeter equipped with an integrating sphere. The same acetone solutions (of absorbance in the range of 0.1–0.2) and thin solid films were used. The measurement was performed in quartz cuvettes with an optical path length of 3 mm. The excitation wavelength of 300 nm was used. The quantum yield study using the integrating sphere is based on the absolute method. It compares the number of emitted photons with the number of photons absorbed by the sample, which allows for omitting the use of a standard with known values of quantum fluorescence yields [58].

#### 3.3.3. Contact Angle Measurement

The measurement of the static contact angle (Θ, with accuracy ±2°) was performed using the DSA goniometer from KRUSS GmbH (Hamburg, Germany) using the sessile drop method at constant room temperature. Films with a carefully selected, smooth surface and two test liquids of different polarities: glycerin and diiodomethane, were used for these studies. Drops with three μL volume were applied to the surface of the samples using a syringe with a needle diameter of 1.5 mm. The contact angle was determined immediately after the drop was applied. Several measurements were made for each specimen. The surface free energy (SFE) was calculated using Young’s equation and the Owens–Wendt method, which assumes that the total surface free energy (SFE) is the sum of two components: polar SFE(P) and dispersive SFE(D) [59]. For analysis of the drop shape, observed with a digital camera, and SFE calculation, the software supplied by the producer was used.

#### 3.3.4. Morphology Analysis by SEM and AFM

The surface morphology of the obtained samples was examined with the scanning electron microscope model 1430 VP by LEO Electron Microscopy Ltd., (Cambridge, UK). A gold layer of about 18 nm thickness was sputtered on the specimens. The images of the internal structure (cross-sections) on brittle fractures of the samples (in liquid nitrogen) were taken. The surface topography and roughness were also examined using a digital instrument multimode nanoscope atomic force microscope. The following roughness parameters were determined from the surface profile: the root mean square roughness—R_q_, arithmetic mean—R_a_, and maximum height within the definition area—R_max_.

#### 3.3.5. Protein Adsorption

The interaction of the proteins: human serum albumin (HSA) and α-acid glycoprotein (AGP) with the chitosan membrane and dye-doped chitosan membranes was investigated using the Jasco FP-800 spectrofluorimeter. A solution of human serum albumin (HSA) with a concentration of 6 μM and α-acid glycoprotein (AGP) with a concentration of 10 μM in phosphate buffer pH = 7.4 and a concentration of 0.05 M was prepared. Square film samples with dimensions of 2 × 2 cm^2^ were immersed in 2 mL of protein solutions and then incubated at 36 °C in a VWR International Thermomixer at 600 rpm. The fluorescence spectra in the wavelength range of 285–400 nm for HSA and 300–400 nm for AGP were recorded at various time intervals. The measurements were repeated three times.

A standard curve was prepared for each of the proteins used to calculate the amount of adsorbed proteins on the obtained composites. The human blood albumin serum standard solution concentration in the phosphate buffer was 9 µM. The HSA solutions with a concentration of 1 to 9 µM were prepared. A calibration curve was obtained by performing spectrofluorimetric measurements at a wavelength of 333 nm, i.e., for the maximum emission intensity. Similarly, a calibration curve of a standard solution of α-acid glycoprotein serum in phosphate buffer (10 µM) was obtained by performing spectrofluorimetric measurements at a wavelength of 329 nm (at maximum emission).

#### 3.3.6. Kinetics of the Dyes’ Release

A piece of the square-shaped film (1 × 1 cm^2^) containing 5 wt. % dye (**I** or **H**) and 20 cm^3^ of methanol were introduced into Eppendorf test tubes. The filled tubes were placed in the thermomixer and shaken at 1000 rpm. Following the shaking, 4 mL of extract were collected to obtain the UV-Vis absorption spectra. A calibration curve was previously designated for methanolic solutions of different dye concentrations (from 0 to 5 mg/L) to quantify the dye released. The standard deviation for the release after 12 h was 1.86 and 1.94 for **H** and **I** dye, respectively.

#### 3.3.7. Mechanical Properties

Mechanical properties of pure chitosan films and chitosan doped with the BODIPY compounds were studied using the Shimadzu EZ-Test E2-LX machine (Shimadzu, Kioto, Japan) based on the ISO standard (ISO 527-2:2012). The samples of each type of film were prepared and cut into a straight strips. Thus, the prepared specimens were clamped between pneumatic holders. The tensile tests were performed at an elongation rate of 20 mm/min. The measurement was repeated at least five times for each sample, and the results were averaged.

## 4. Conclusions

The photosensitive composites of BODIPY with chitosan were prepared by physical mixing and characterized by spectroscopic and scanning electron microscopy methods. Films have flat, smooth surfaces, while the internal structure is heterogeneous. FTIR spectroscopy proves the intermolecular interactions between the dyes and biopolymer, which can lead to aggregation, not only in a solid state. The introduction of BODIPY to chitosan causes a slight increase in the hydrophilicity, which positively affects their gradual release in time and human protein adsorption.

BODIPY-modified chitosan becomes fluorescent and exhibits a bathochromic shift of the absorption and emission bands relative to the original dyes, which is advantageous for potential medical applications [39]. The Stokes shifts are much higher in the films than those in the solutions. An additional therapeutic effect may be exerted by the phthalimide substituent in the meso position, which, due to its large volume, hinders the formation of aggregates. The previously confirmed antibacterial effect of the BODIPY is an additional benefit of this system [26].

Iodine introduced into the BODIPY structure causes a decrease in the fluorescence quantum yield, and this trend also concerns the **CS-H** and **CS-I** compositions (films and solutions).

The fluorescence, good adsorption, gradual dye release, and mechanical properties of the obtained materials allow us to propose them for biological sensing and imaging or for the production of patches that deliver a photosensitizer to the affected skin in PDT. However, before introducing these materials into medical practice, it would be necessary to perform biocompatibility tests in vitro and in vivo.

By gaining the ability to fluoresce, the CS-BODIPY system does not lose the valuable physicochemical properties of the macromolecular compounds. Thin, durable films can be obtained, which facilitates the application on the skin surface. It should be added that the dyes themselves exist in powder form and do not have film-forming properties. The simplicity of obtaining chitosan-BODIPY films suggests potential benefits in the implementation in practice.

## Figures and Tables

**Table 1:** Maxima of the main absorption bands in **I** and **H** dyes, FTIR-ATR spectra, and their assignment.

IR Band Position, cm^−1^		Assignment [29,30,31,32]
I	H	
3044	3083	C-H aromatic and heterocyclic, stretching
2928	2947	C-H aliphatic, stretching
1690	1710	C=O (amide I) stretching
1507	1489	N-H (amide II), C=C aromatic,_._ stretching
1380	1387	C-H deformation, B-N stretching
1163	1177	C-O stretching
1095	1054	C-O stretching
990	983	C-C stretching
-	822	C-H deformation
718	712	C-H, B-N deformation
523	-	C-I stretching

**Table 2:** The main absorption bands (cm^−1^) in the FTIR spectra of **CS**, **CS**-**I,** and **CS**-**H** composites and their assignment.

IR Band Position, cm^−1^			Assignment [33,34,35]
CS	CS-I	CS-H	
3367	3272	3277	O-H/N-H stretching
2924, 2879	2930, 2885	2928, 2875	C-H stretching (symmetric and asymmetric)
1650	1645 (arm of amide II band)	1663 (arm of amide II band)	C=O (amide I) stretching
1563	1566	1539	N-H (amide II) stretching
1409	1397	1393	C-H/C-N deformation
1080	1057	1059	C-O-C deformation
899	barely visible	barely visible	C-H bending saccharide ring, out of the plane
655	632	643	C-H deformation
-	578	-	C-I stretching

**Table 3:** Spectral characteristic of the studied samples: maxima of absorption (λ_abs_) and emission (λ_em_), stokes shifts (Δλ), and fluorescence quantum yield (Φ_F_). Spectra of **I** and **H** dyes were recorded in acetone solution; **CS-I** and **CS-H** in aqueous acetic acid-acetone (3:1) solution.

Sample	λ_abs_, nm	λ_em_, nm	Δλ, nm	Φ_F_, %
**I** solution	529	553	24	2.60
**CS-I** solution	545	571	26	1.95
**CS-I** film	545	603	58	0.99
**H** solution	498	513	15	19.4
**CS-H** solution	522	546/570	24/48	2.97
**CS-H** film	522	570/602	48/80	2.45

**Table 4:** The mean values of the contact angle, surface free energy, and its polar and dispersion components for pristine chitosan and chitosan doped with dyes.

**Sample**	**Contact Angle (θ,°)**		**Surface Free Energy (mJ/m^2^)**		
	**Test Liquid**				
	**Glycerin**	**Diiodomethane**	**SFE**	**SFE(D)**	**SFE(P)**
**CS**	82.0	56.0	30.70	27.46	3.23
**CS-I**	72.9	61.1	30.71	22.07	8.64
**CS-H**	78.4	58.8	30.00	24.76	5.24

**Sample**

**Contact Angle** **(θ,°)**

**Surface Free Energy** **(mJ/m^2^)**

**Test Liquid**

**Glycerin**

**Diiodomethane**

**SFE**

**SFE(D)**

**SFE(P)**

**Table 5:** Roughness parameters for the chitosan film and chitosan doped with **I** and **H** dyes for the scan area 1 × 1 µm^2^ and 10 × 10 µm^2^.

Sample	Roughness Parameters [nm] for 1 × 1 µm^2^ and 10 × 10 µm^2^		
	R_q_	R_a_	R_max_
**CS**	1.40/4.30	1.08/2.94	11.7/54.0
**CS-I**	1.66/1.98	1.31/1.51	15.1/41.8
**CS-H**	3.43/26.1	2.56/13.2	26.7/477

**Table 6:** Mechanical properties of the chitosan film and chitosan doped with **I** and **H** BODIPY.

Sample	Breaking Stress, MPa	Ultimate Elongation, %	Young’s Modulus, MPa
**CS**	28.6 ± 0.8	2.9 ± 0.3	1169 ± 137
**CS-I**	22.5 ± 4.8	1.8 ± 0.2	423 ± 53
**CS-H**	8.6 ± 5.0	2.8 ± 0.8	767 ± 64

## Data Availability

Not applicable.
